# Interpreting the Entire Connectivity of Individual Neurons in Micropatterned Neural Culture With an Integrated Connectome Analyzer of a Neuronal Network (iCANN)

**DOI:** 10.3389/fnana.2021.746057

**Published:** 2021-10-20

**Authors:** June Hoan Kim, Jae Ryun Ryu, Boram Lee, Uikyu Chae, Jong Wan Son, Bae Ho Park, Il-Joo Cho, Woong Sun

**Affiliations:** ^1^Department of Anatomy, Korea University College of Medicine, Seoul, South Korea; ^2^Center for BioMicrosystems, Brain Science Institute, Korea Institute of Science and Technology (KIST), Seoul, South Korea; ^3^Division of Quantum Phases and Devices, Department of Physics, Konkuk University, Seoul, South Korea

**Keywords:** network, connectome, connectivity, axon guidance, micropattern

## Abstract

The function of a neural circuit can be determined by the following: (1) characteristics of individual neurons composing the circuit, (2) their distinct connection structure, and (3) their neural circuit activity. However, prior research on correlations between these three factors revealed many limitations. In particular, profiling and modeling of the connectivity of complex neural circuits at the cellular level are highly challenging. To reduce the burden of the analysis, we suggest a new approach with simplification of the neural connection in an array of honeycomb patterns on 2D, using a microcontact printing technique. Through a series of guided neuronal growths in defined honeycomb patterns, a simplified neuronal circuit was achieved. Our approach allowed us to obtain the whole network connectivity at cellular resolution using a combination of stochastic multicolor labeling via viral transfection. Therefore, we were able to identify several types of hub neurons with distinct connectivity features. We also compared the structural differences between different circuits using three-node motif analysis. This new model system, iCANN, is the first experimental model of neural computation at the cellular level, providing neuronal circuit structures for the study of the relationship between anatomical structure and function of the neuronal network.

## Introduction

The brain is a highly complex, yet incredibly well-organized, network of neurons that performs a variety of functions, from basic life support to high-order information processing. Brain circuits perform computations through a combination of basic elements and their connections (Silver, 2010). These neurons are the fundamental units of information processing, with various characteristics distinguished by their neuronal types (Kepecs and Fishell, 2014; Jiang et al., 2015). Therefore, investigating the features of individual neurons and their connectivity properties is crucial for understanding the mechanisms underlying neural computation. Neurons can be classified based on their morphological, electrophysiological, and molecular features (Pelkey et al., 2017), and different neuronal types with different connectivity properties appear to play a role in the formation of neural networks (Kepecs and Fishell, 2014; Jiang et al., 2015). For example, some early generated hippocampal GABAergic interneurons make many synaptic connections and become hub neurons in the early postnatal stages, orchestrating very important synchronous activity for circuit maturation (Bonifazi et al., 2009; Picardo et al., 2011). In addition, network modules, called motifs, appear to be established owing to the unique connectivity of specific neurons, as suggested in many studies (Sporns and Kotter, 2004; Womelsdorf et al., 2014; Tremblay et al., 2016; English et al., 2017).

Identification of general rules for the establishment of motifs is one of the goals of computational neuroscience, thereby increasing knowledge of complex network phenomena (Womelsdorf et al., 2014; Tremblay et al., 2016; English et al., 2017). The development of new technologies, such as high-resolution multi-channel *in vivo* recordings and connectomics approaches, allows for ongoing large-scale brain mapping projects for inferring monosynaptic transmission and studying motifs in animal models (Wu et al., 2015; Gal et al., 2017; Jun et al., 2017; Shin et al., 2021; Steinmetz et al., 2021). Owing to large-scale funding, as well as the development of various technical approaches, a significant amount of connectome data across different scales and different species have been collected and shared (Sporns et al., 2005; Helmstaedter, 2013; Oh et al., 2014; Kunst et al., 2019; Majka et al., 2020). By analyzing the connectome data, the structural characteristics of the brain network have been reported (Ercsey-Ravasz et al., 2013; Zingg et al., 2014; Theodoni et al., 2021). For example, long-tailed distribution of cortical network, economic wiring principles in neural networks, hierarchical structures of cortical and thalamic modules, well-balanced spatial distribution of excitatory and inhibitory synapses in the mammalian cortex, and hub structure of mouse brain with voxel resolution have been identified (Schroter et al., 2017; Harris et al., 2019; Coletta et al., 2020; Iascone et al., 2020). In addition to structural connectivity in neural networks, functional connectivity has also been investigated (Alivisatos et al., 2012). A step toward finding general rules in network structure and function, small-size motifs (M = 3, 4) have been analyzed in structural and/or functional connection maps (Sporns and Kotter, 2004; Gal et al., 2017; Del Ferraro et al., 2018; Turner et al., 2020). However, obtaining the entire connection map for integrating structural and functional networks is a highly challenging endeavor, and revolutionized technology is required.

The simplicity and ease of experimental manipulation made *in vitro* neuronal culture make it a preferred method for studying the network activity of neurons. Multi-electrode array (MEA) recording systems are well suited for this goal and have been widely used in many studies to investigate basic properties of neuronal networks, such as synchronized activity, oscillatory behavior of neuronal population, and learning and memory at the network scale (Wagenaar et al., 2005; Choi et al., 2012; Kim et al., 2014). However, for good signal detection, MEAs require densely cultured neurons, increasing the difficulty in analyzing the structural connections of neurons. Therefore, one of the accessible approaches is micropatterning, which prints patterns on a culture substrate with cell-adhesive or -repulsive reagents. The growth of primary neurons on micropatterns can be precisely controlled, making the neural circuits more desirable (Boehler et al., 2012; Marconi et al., 2012). For example, we previously reported that the microprinting of a repulsive signal, semaphorin3F (Sema3F), could spatially confine the synaptic area by simplifying the growth responses of axons and dendrites (Ryu et al., 2016). More importantly, this system can easily be combined with the MEA system to study functional network activities (Marconi et al., 2012; Ryu et al., 2016).

In this study, we attempted to establish a new research platform that enables comprehensive analysis of a closed neural circuit, reconstructed with the entire synaptic connections of individual neurons. Therefore, we optimized the culture conditions to identify individual neurons on micropatterned substrates using stochastic multicolor cell labeling techniques (Chan et al., 2017). Our initial attempts demonstrated that it is feasible to reconstruct and analyze cultured neuronal networks by tracking all the synaptic connections between individual neurons.

## Materials and Methods

The entire process from cell culture to network analysis is shown in Supplementary Figure 1.

### Stamp Fabrication and Microcontact Printing

A stamp mold for printing axon guidance reagents was fabricated on a silicon wafer with standard photolithography techniques using negative photoresist SU-8 2010 (Microchem, United States), and molding polydimethoxysilane (PDMS) into a stamp through curing overnight at 60^*o*^C, as previously described (Joo et al., 2015; Ryu et al., 2016; Ryu et al., 2019) (Supplementary Figure 2). A poly-l-lysine (1 mg/mL) coated coverslip was required for patterning. The 10-μg/mL Semaphorin 3F (3237-S3, R & D Systems) ink was poured onto the surface of a stamp for 1 h at 37^*o*^C. The stamps were rinsed with distilled water and air-dried. The ink-loaded stamp was placed on the PLL-coated coverslips for printing and was slightly pressed using a 20-g weight for 10 min. The printed coverslip was ready for culture after rinsing with Dulbecco’s phosphate-buffered saline.

### Primary Neuronal Culture

Hippocampi were isolated from E18 embryonic Sprague–Dawley rats (timed-mated) and dissociated into single cells, as previously described (Ryu et al., 2016; Ryu et al., 2019). Neural cells in MEM-based culture media (10% FBS, 1% penicillin–streptomycin [PS], 1% N2 supplement, and 3.6 mg/mL glucose) were seeded in culture dishes at a density of 39.5–78.9 cells/mm^2^. Culture media were replaced with incomplete culture media (ICM) (2% B-27 supplement, 0.5% L-glutamax, and 1% PS in Neurobasal media) 3 h after plating. CultureOne^TM^ supplement (A3320201, Gibco) was added to the ICM to obtain pure neuron culture to avoid micropatterns covered by astrocytes. CultureOne supplement drives the differentiation of neuroprogenitor cells to neurons without side effects on neuronal morphology (Thermofisherscientific, 2017). B-27 supplement was replaced with B-27plus supplement (A3582801, Gibco) to improve the survival rate of cultured neurons. Animal care and euthanasia protocols followed the Korea University guidelines and were approved by the Korea University Institutional Animal Care and Use Committee.

### Stochastic Multicolor Labeling, Immunostaining, and Imaging

Recombinant adeno-associated viruses (AAVs) were generated through transfection of HEK293T cells using polyethylenimine (PEI) and purified using ultracentrifugation (Ti70 rotor, Beckman) over iodixanol (D1556, Sigma-Aldrich), as described in Challis et al. (2019). The four-vector mix of pAAV-hSyn1-tTA (Addgene plasmid # 99119), pAAV-TRE-mRuby2 (Addgene plasmid # 99114), pAAV-TRE-EGFP (Addgene plasmid # 89875), and pAAV-TRE-mTurquoise2 (Addgene plasmid # 99113) was packaged into the AAV-PHP.eB capsid (CLOVER, Caltech). The pAAV-hSyn1-tTA, pAAV-TRE-mTurquoise2, pAAV-TRE-mRuby2, pAAV-PHP.eB capsids were provided by Viviana Gradinaru, and pAAV-TRE-EGFP was provided by Hyungbae Kwon. Cultured neurons were transfected with 0.5–1.5 × 10^13^ vg/well (for 15,000 cells) at days in vitro (DIV) 3–7. Cells were fixed with 4% paraformaldehyde and 4% sucrose in 0.1 M phosphate buffer (PBS) (pH 7.4) at DIV 21, as previously described (Ryu et al., 2016), and were mounted on microscope slides after immunostaining when necessary. For immunocytochemistry, the fixed samples were incubated with primary antibodies in blocking solution (3% bovine serum albumin, 0.2% Triton X-100 in Tris-buffered saline (TBS) overnight at 4^*o*^C after 30 min blocking. Samples were washed three times with 0.05% Triton X-100 in TBS for 10 min each under gently shaking, and then stained with appropriate secondary antibodies for 30 min. Antibodies against GFAP (rabbit, 1:10000, z0334, DAKO), MAP2 (chick, 1:500, AB5543, Millipore), and Tuj1 (mouse, 1:2000, T8660, Sigma-Aldrich) were used. The slides were imaged with a slidescanner (Axio Scan.Z1, Zeiss) using a 10× objective lens or a confocal microscope (TCS SP8, Leica) using a 63× objective lens for imaging synapses.

### Image Processing and Data Analysis: ImageJ, MATLAB Codes

(Image rotation) Initially, neuron images with patterns were rotated to facilitate image processing. The rotational angle was obtained using Fast Fourier Transform (FFT) on a pattern image. FFT is useful for identifying repetitive patterns and determining their alignment angle.

(Pattern detection) 2D cross correlation between the rotated pattern image and a template pattern image was calculated. Finally, the positions of individual patterns were obtained by detecting circles from the 2D cross correlation image circles and were indexed.

(Cell, dendrite, and axon segmentation) Cells, dendrites, and axons were manually segmented using in-house GUI MATLAB codes. During segmentation, the cell image with a pattern was scanned through zooming and shifting of the field of view. To discriminate individual cells by color, we adjusted color balance between three colors: red, green blue, and brightness of the pattern in each field of view. The positions of cellular components of each neuron were obtained by placing the cursor on the pattern with the desired cellular components, and on the keyboard pressing the “c” button for cells, “d” button for dendrites, and “a” button for axons. The pattern positions with dendrites or axons were matched to the pattern indices. As all synapses were formed in patterns, the positions of patterns with dendrites and axons annotated with a cell index were sufficient for reading synaptic connections.

(Network analysis) Based on our observations, two neurons considered synaptically connected when the dendrites of one cell met the axons of the other cell within the pattern. Co-location of Synapsin and PSD95 signals was observed in most patterns, where the axons of one neuron and the dendrites of another neuron coexisted. A table of connected cell pairs was imported using Cytoscape (Shannon et al., 2003) to visualize the reconstructed networks and analyze degree, eccentricity, and centralities. The mathematical definitions of the analytic terms are:

In-degree of a node (k_*in*_): the number of edges to a node from other nodes.
Out-degree of a node (k_*out*_): the number of edges from a node to other nodes.
Density *D* = *k*/*N*,
where *k* is degree and *N* is number of nodes
Eccentricity ***e***(***n***) = **max**_***m***∈***N***_ ***d***(***n***, ***m***),
where the distance ***d***(***m***, ***n***) between two nodes *m* and *n* is defined as the length of the shortest directed path from ***m*** to ***n***.
Closeness centrality ${\text{C}}_{C}\,\left(n\right)=\frac{N}{\sum_{m}d\,\left(n,m\right)}$ (0 for an isolated node),
Betweenness centrality ${\text{C}}_{B}\,\left(n\right)=\sum_{s\neq n\neq t}\frac{\sigma_{\mathrm{st}}\,\left(n\right)}{\sigma_{\mathrm{st}}}$,

where ***s*** and ***t*** are nodes in the network different from ***n***, **σ**_***st***_ is the total number of shortest paths from ***s*** to ***t***, and **σ**_***st***_(***n***) is the number of shortest paths from ***s*** to ***t*** passing through ***n***.

(Three-node motif analysis) We selected triplet node sets from the adjunct matrix and investigated which classes were included among 13 classes of three-node motifs using in-house MATLAB codes.

## Results

### Micropatterning and Neuronal Growth Patterns

**FIGURE 1:**
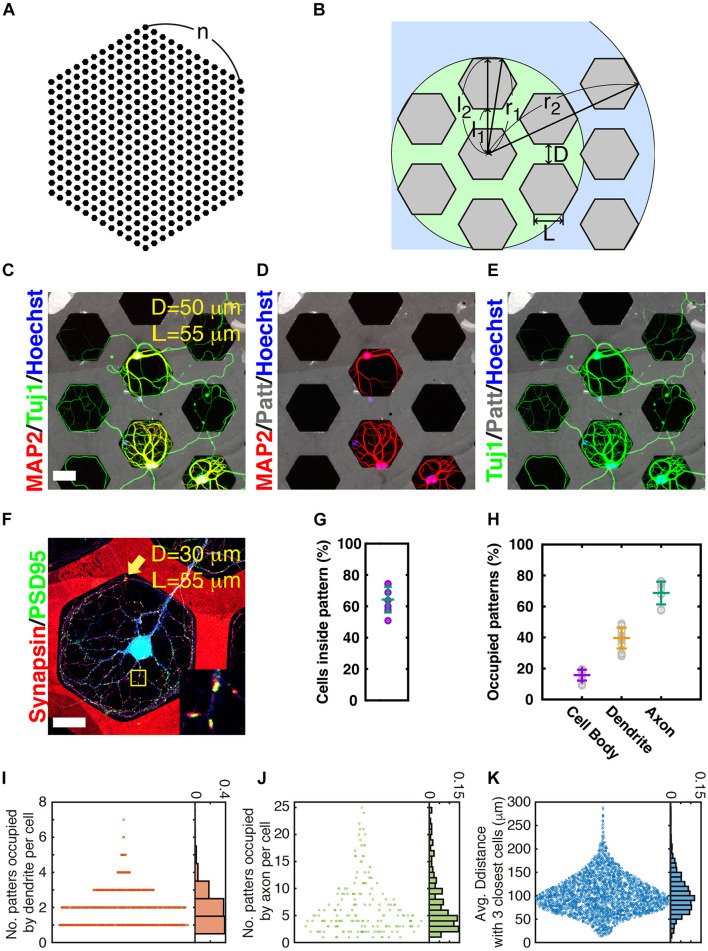
Neuronal growth on micropatterns. **(A)** Honeycomb pattern. **(B)** Dimension of honeycomb pattern. L: edge length of hexagon, D: gap between hexagonal patterns, r_1_: radius of a circle covering first nearest neighbors, r_2_: radius of a circle covering second nearest neighbors, l_1_: length from the center of a hexagonal pattern to the closest edge of a nearest neighboring pattern, l_2_: length from the center of a hexagonal pattern to the farthest edge of a nearest neighboring pattern. **(C–E)** Example image of neurons grown on honeycomb patterns (gray, edge [L]: 55 μm, gap [D]: 50 μm) for 21 days *in vitro*. (Scale bar: 50 μm). Dendrites stained using MAP2 and honeycomb patterns, and neuritis stained using Tuj1. **(F)** Representative image of a receptive field (synapse formation zone). Red: synapsin (presynaptic) and Green: PSD95 (postsynaptic). Blue: neurons. Note that patterns are also marked with red fluorescence. Inset: enlarged image of yellow box. (Edge [L]: 55 μm, gap [D]: 30 μm) (scale bar: 50 μm) **(G)** Probability of cells inside patterns. Error bars: average and SD. (*N* = 4811 cells from 7 culture samples). **(H)** Proportion of patterns occupied by cell body, dendrite, and axon with average and SD values. (N: 9436 cells, 32916 patterns, 14 culture samples). **(I)** Number of patterns occupied by dendrites of individual neurons and normalized histogram (probability). (N: 1360 cells from 2 culture samples). **(J)** Number of patterns occupied by axons of individual neurons and normalized histogram (probability). (N: 314 cells from 9 culture samples). **(K)** Average distance from a cell to the three closest neurons. Histogram was normalized as probability [same samples with panel **(I)**].

Microcontact printing is a simple technique for micrometer-level processing of the surface of a culture substrate. In this study, the pattern of permissive PLL dots surrounded by Sema3F for repulsive axon guidance cues was used. The Sema3F-negative PLL dot array patterns allow the allocation of synapses in the dot pattern compartmentalized from long, complex growing axons (Ryu et al., 2016). In addition, axons can grow to be very long, but they cannot extend very far from the cell body owing to entrapment of the PLL dots, which facilitates obtaining simplified artificial neuronal circuits. First, we observed the ways in which axons interacted with Sema3F-negative dot array patterns (Supplementary Figure 3). During the maturation of the neural circuits on micropatterns, axons grew radially from the cell body and where the axon terminal met the interface between Sema3F and PLL zones, it grew along the interface line (0–120 min in Supplementary Figure 3). Axons often escaped from the permissive to repulsive zone at the sharp angular point of the triangle pattern (630 min in Supplementary Figure 3) and extended into a neighboring pattern (990 min in Supplementary Figure 3). We speculated that the shape of pattern with a smaller angle made it easier to escape axons from the pattern. Conversely, larger-angled patterns could better capture axons inside the pattern than smaller-angled ones. As a results, the distances from the cell body to the maximal extension of the axon in hexagon (120^*o*^) and square (90^*o*^) were significantly shorter than triangle (60^*o*^) (Supplementary Figure 4). The difference between the square pattern and the hexagonal pattern is not very significant, but the hexagonal pattern was empirically easier to analyze. Based on these observations, we adopted a honeycomb pattern to reduce the chance of maintaining axons within one hexagonal pattern (Figures 1A,B). Maturing patterns of DIV 21 neurons in honeycomb patterns are shown in Figures 1C–E. The rate of the cells inside the patterns was 64.3 ± 8.2% (Figure 1G). Though both axons and dendrites remained inside hexagonal patterns (Figures 1C–E) under our experimental conditions, axons often migrated to other hexagonal patterns. In addition, synapses were well established inside the permissive area, as previously reported (Ryu et al., 2016) (Figure 1F). An axon intruded into a receptive pattern area (RPA) of blue colored cells (yellow arrow in Figure 1F) and formed synaptic connections with blue cells. The co-location of synapsin (red) and PSD95 (green) signals is shown in the inset of Figure 1F. We found that >90% of hexagonal patterns with both axons and dendrites exhibited synapse formation, suggesting that most of the spatial contact resulted in synapse formation. As we expected, using hexagonal patterns, the complexity of neuronal network was obviously reduced suitable for the further quantitative analysis (Supplementary Figure 5).

To estimate the range of diversity of artificial neural circuits, we quantified the occupancy of patterns by cell bodies, dendrites, and axons from 14 different samples, cultured on hexagonal patterns with 55-μm edge length (L) of a hexagon and a 70-μm gap between hexagons (D). The occupancy values were 9.0–19.5% (average ± SD: 15.7 ± 3.4%) for cell bodies, 28.0–49.0% (average ± SD: 39.6 ± 6.7%) for dendrites, and 57.4–76.4% (average ± SD: 68.7 ± 7.3%) for axons (Figure 1H). We counted patterns occupied by the dendrite/axon of each neuron to assess the coverage of dendrites and axons of each neuron (Figures 1I,J). The number of patterns occupied by the dendrites of a cell is equivalent to the number of RPAs. This does not mean that the number of RPAs is the capacity of synaptic connections, but a large number (area) of RPAs provide a greater chance of building synaptic connections. A total of 76.2% of neurons formed one or two RPAs (Figure 1I). The cell outside pattern tended to make a more RPA owing to its easy accessibility to many neighboring patterns. A neuron occupied an average of 6.7 patterns with its axon. The number of patterns occupied by axons per cell varied from 1 to 25, and neurons tended to cover 2–4 patterns; meanwhile, most neurons (80.8%) covered fewer than 10 patterns (Figure 1J). Collaboration of axon coverage of each cell and the distance between neighboring cells contributed to the complexity of the circuit. The average distance from the cell to the three closest neighboring cells was determined (Figure 1K). The average distance of 59.5% was in the range 60–120 μm, indicating that neurons with this patterning condition had a high chance of reaching synaptic partners, since neurons had neighboring cells in the nearest hexagonal patterns. The distances from the center of a hexagon to the closest or furthest edge of the neighboring hexagon (l_1_ and l_2_ in Figure 1B) were 117.6/212.8 μm, respectively, in this pattern condition; the edge of the hexagon (L) was 55 μm, and the gap between hexagons (D) was 70 μm.

### Network Reconstruction by Tracing Connectivity of Neurons

**FIGURE 2:**
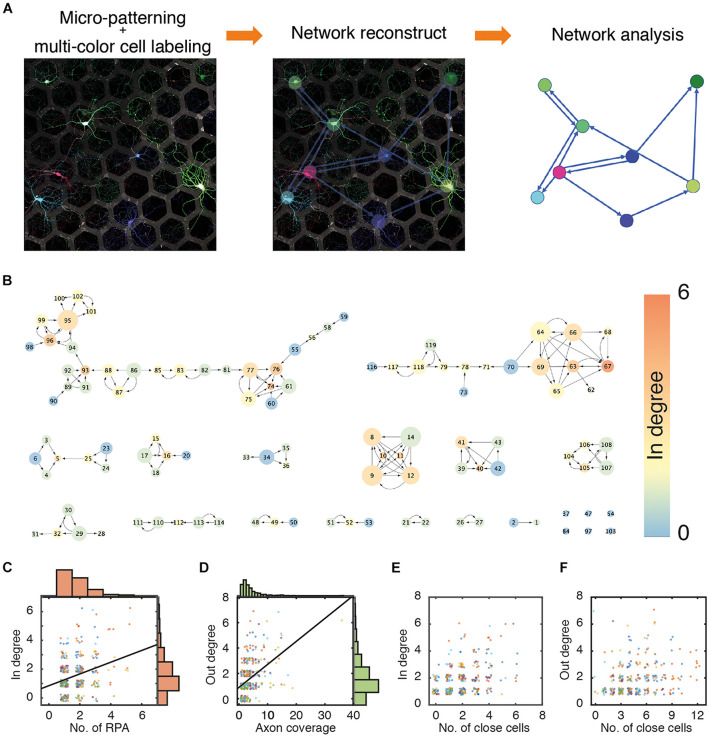
Network reconstruction. **(A)** An example of multi-colored labeled neuron culture and reconstruction of a neuronal network. Edge (L): 55 μm. Gap (D): 30 μm. **(B)** A representative network of a culture neuronal circuit. Color of a node and size of a node indicate in-degree and out-degree, respectively. **(C)** Scatter plots and histograms of a number of patterns occupied by dendrite of individual neurons (RPA) and their in-degree values. Black line (linear fit); *y* = 0.4363×*x*+0.8179, *R*^2^ = 0.09573. **(D)** Scatter plots and histograms of a number of patterns covered by axons from individual neurons (axon coverage) and out-degree values. Black line (linear fit) *y* = 0.1804×*x*+0.0.9031, *R*^2^ = 0.2178. **(E)** Scatter plots of numbers of neighboring neurons in first nearest neighboring hexagonal patterns and in-degree values. **(F)** Scatter plots of numbers of neighboring neurons in first and second nearest neighboring patterns and out-degree values. The data points were jittered in the 0.5 range to avoid overlap. (N: 434 cells from 6 culture samples).

The stochastic multicolor labeling technique endows cells with a color that is randomly composed of several colors, such as red, green, and blue. Recently, since the introduction of Brainbow, several improved techniques have been suggested (Livet et al., 2007; Cai et al., 2013; Chan et al., 2017). A two-component inducer system using the tetracycline (tet)-inducible system is reportedly better for various color expressions (Chan et al., 2017). In addition, the PHP.eB serotype of AAVs efficiently produced higher CNS transduction than other serotypes in *in vivo* studies (Chan et al., 2017; Dayton et al., 2018; Mathiesen et al., 2020). This multicolor labeling system was applied to the primary culture of pure neurons, and individual neurons were easily discriminated from each other (see Figure 2A). However, the fluorescence intensities in different compartments of the same cell varied, and local aggregation of similar colors through global optimization of fluorescence intensities often reduced the fidelity of cell identification. To precisely mark the cell-to-cell connectivity, we developed MATLAB codes, enabling the manual labeling of cell bodies, dendrites, and axons by locally adjusting the color balance and recording their pattern position, as described in the Materials and Methods section of this manuscript. Through post-processing, most connectivity in the *in vitro* neural circuits could be interpreted at cellular resolution. A representative network reconstructed from an image of cultured neurons in 397 hexagonal patterns (*n* = 12) is shown in Figure 2B. The color of a node indicates the in-degree value of a node, and the size of a node reflects the out-degree value of the node. The direction of the arrow is from the presynaptic neurons to postsynaptic neurons. The network consisted of 15 large and small network segments and 6 isolated nodes. The degree distribution of the six different cultures of neurons is shown in Supplementary Figure 5.

Surprisingly, the rules of connection between neurons did not seem straightforward despite the neural circuits established on micropatterns being very simple; indeed, there was only a weak correlation between the number of RPAs of a cell and in-degree values of that cell (Figure 2C), since axons from different neurons can penetrate the same RPA, as previously mentioned. Similarly, axon coverage of a cell did not correlate with the out-degree value of that cell (Figure 2D). The reason appears to be that even if axons grow long and cover a wide area, the cell cannot synapse with other cells without any target cell in the growth area of the axon. Cells in neighboring patterns can be candidates for pre/post-synaptic neurons. However, the number of cells in the nearest neighboring patterns (in a circle with radius r_1_; Figure 1B) did not have a significant correlation with the in-degree value (Figure 2E), and the number of cells in patterns in the range of the next nearest neighboring patterns (in a circle with radius r_2_; see Figure 1B) did not correlate with the out-degree value (Figure 2F). Therefore, the formation of synaptic connections in the micropatterns did not simply depend on the growth range of the cells or the number of neighboring cells (cell density).

### Network Analysis

**FIGURE 3:**
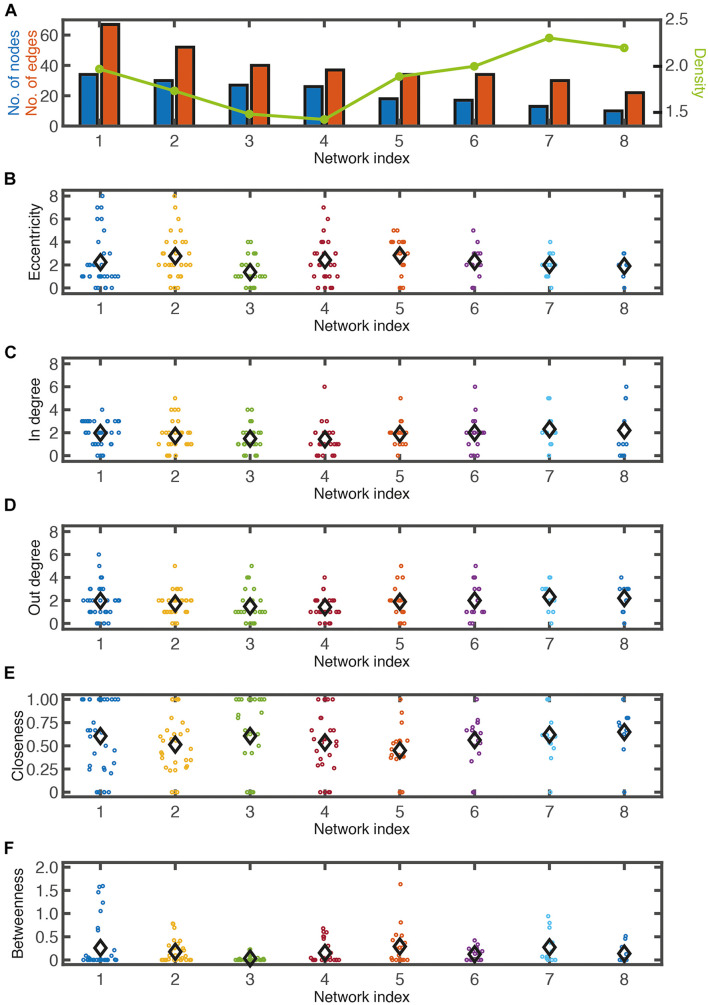
Network analysis. **(A)** Number of node (blue bars), number of edge (red bars), and network density (green line) from eight different networks shown in this figure. Eccentricity **(B)**, in-degree **(C)**, out-degree **(D)**, closeness centrality **(E)**, betweenness centrality **(F)** of each node in eight networks. Black diamond symbols indicate averages.

**FIGURE 4:**
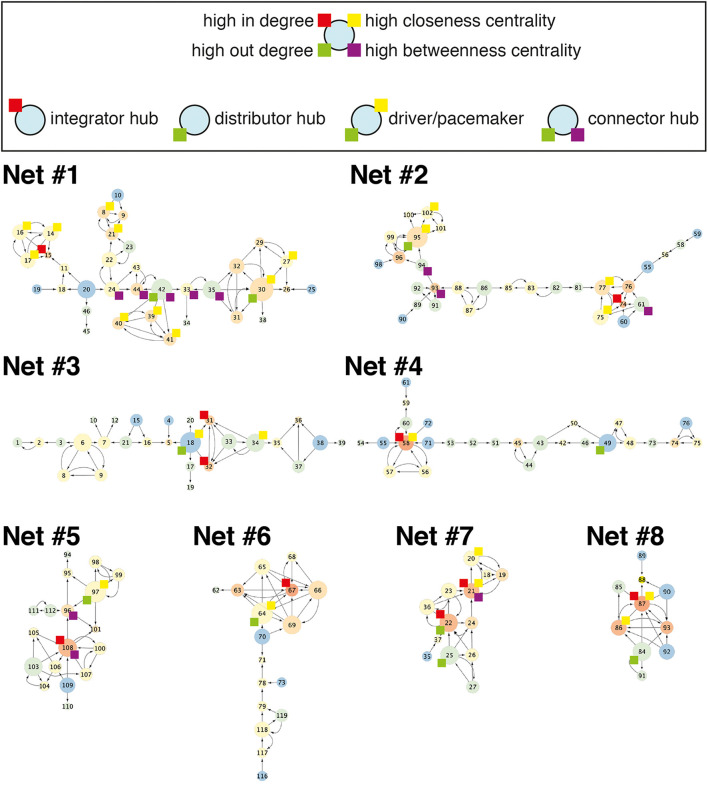
Example networks. Eight different networks from six culture samples. Color of a node and size of a node indicate in-degree and out-degree, respectively. Nodes with high in-degree, out-degree, closeness centrality, and betweenness centrality were tagged with a box of colors: red, green, yellow, and purple, respectively. Net #2 is the same network in Figure 2B.

Networks of various sizes and structures were observed under the same micropatterning conditions. To determine the characteristics of the reconstructed network from cultured neuronal circuits, a graph theory analysis was applied. Though the analysis of a neuron’s wiring pattern alone cannot explain the functional roles of the neuron, it offers the possibility of a functional role of the neuron in the network. Therefore, it is necessary to understand the anatomical structure of neuronal networks in order to understand their function. The number of nodes and edges, eccentricity, in-degree, out-degree, closeness centrality, and betweenness centrality were analyzed in eight different network segments from six different samples cultured on honeycomb patterns (L = 55 μm, D = 70 μm) (Figure 3; also shown in Figure 4). Regardless of the network sizes and number of nodes/edges or eccentricities, the density values of the networks were diverse (line graph in Figures 3A,B). The eccentricity of a node is the distance from the node to the farthest node, and the minimum and maximum values of eccentricity are defined as the radius and diameter of the network, respectively. Thus, the distribution of eccentricity provides an estimation of the network size. The network diameters of the sample networks ranged from 3 to 8 (Figure 3B). In/out-degree values are important basic indicators of local centrality in a directed network. Nodes with more than 4 or 5 in/out degrees depend on the networks (Figures 3C,D) and are tagged by red and green, respectively (see Figure 4). While nodes with a large in-degree can integrate information from neighboring nodes, nodes with a large out-degree can constitute information sources by distributing information to neighbors. These large in/out-degree cells are defined as local hubs, and hub cells are readily identifiable. For example, Cell #74 in Net #2 (in-degree 5), Cell #58 in Net #4 (in-degree 6), and Cell #67 in Net #6 (in-degree 6) are representative examples of the local (integrator) hub (Figure 4). Net #1-Cell #30 (out-degree 6), Net #2-Cell #95 (out-degree 5), and Net #3-Cell #18 (out-degree 5) are examples of the local (distributor) hub (Figure 4). To investigate global efficiency or influence, closeness centrality and betweenness centrality were analyzed (Figures 3E,F). For example, Cell #30 in Net #1 in Figure 4 had a high out-degree value (6) and high closeness centrality (1). These results indicate that Cell #30 could drive many local neighbor cells (high out-degree), while simultaneously having easy access to other nodes (high closeness centrality). We empirically defined cells with these features as pacemaker, or driver, cells. Information from a source (pacemaker) travels through the network. During information transmission, important nodes have high betweenness centrality or stress values. The nodes with high betweenness centrality, tagged in purple in Figure 4, are recruited in the shortest path lengths among the nodes, indicating that heavy information flows through that node. Therefore, these cells may play an important role in information transmission or serve as bottlenecks. In Net #1, Cells #24, #44, #42, #33, and #35 also exhibited high betweenness centrality. Among these, the route from Cell #24 to #35 appears as the main path among local networks, such as an express highway connecting cities (Figure 4). In addition, Cell #42 has a high out-degree value (4), and it can be regarded as a connector hub that connects the local network modules (Bullmore and Sporns, 2009). Therefore, information from Cell #30 flows to Cell #15 via the connecting route, including the Cell #42 connector hub. Cell #15 can be readout of Net #1, since information in Net #1 gathers in Cell #15.

**FIGURE 5:**
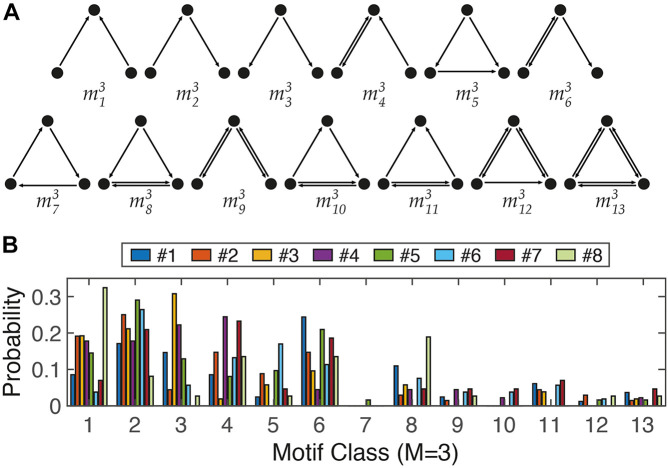
Three-node motifs analysis. **(A)** The 13 different ways (classes) to connect three nodes in a directed network. **(B)** Distribution profile of 13 motifs from 8 networks in Figure 4.

It is believed that structural motifs have functional significance owing to over-representation of a certain motif in a network. In particular, in the study of the relationship between the structure of a neural circuit and its function (Sporns and Kotter, 2004; Tremblay et al., 2016; English et al., 2017), analysis of structural motifs can be a useful quantitative measure. Analysis of small motifs can quantify interactions between neighboring nodes (Bullmore and Sporns, 2009). Interestingly, common characteristics were found in the analysis of motif profiles across different species/networks. For example, classes 4, 6, and 9 of three-node motifs were frequently observed in four different functional networks of the brain (macaque visual cortex, macaque cortex, fine-grained macaque cortex, and cat cortex) (Sporns and Kotter, 2004; Gollo et al., 2014); meanwhile, class 7 was very rare across the five brain networks (*C. elegans*, macaque visual cortex, macaque cortex, fine-grained macaque cortex, and cat cortex). We analyzed size 3 motifs (M3) in our simplified neuronal circuits and attempted to identify common features across different neural circuits as well as circuit-dependent characteristics. The difference in distribution of motif classes between neural networks and random, or lattice, structures was apparent (Sporns and Kotter, 2004). In general, classes 7–13 of the 13 different three-node motifs occurred less frequently. In particular, class 7 was very rare. Surprisingly, this may be an indication of the existence of a ubiquitous wiring rule in neural networks, avoiding the recurrent relation of the three nodes. However, eight networks showed clear differences in the three-node motif profiles (Figure 5B). For example, the representative types of neural circuit structures are the feedforward motif (m^3^_5_) and feedback motif (m^3^_6_) (Tremblay et al., 2016). While the feedback structure (m^3^_6_) was frequently observed in Nets #1, #5, and #7, the feedforward structure (m^3^_5_) was more dominant in Net #6. Therefore, motif analysis can be considered a useful tool for quantitatively measuring network structures, such as using basis vectors in linear algebra.

## Discussion

By combining synapse compartmentalization through micropatterning with stochastic multicolor labeling, we developed a high-resolution neural circuit analysis platform. Simplified *in vitro* neural circuits were built by controlling axonal growth and the synapse formation domain with micropatterns of the repulsive axon guidance protein Sema3F on a PLL-coated culture substrate. Individual neurons were labeled with different colors, enabling the interpretation of the entire connection in the neural circuit. Neuronal networks were well reconstructed using the connection information. Neurons with distinctive properties were discovered using graph analysis of neural circuits. Furthermore, our results show the potential of the structural motif as a quantitative tool to analyze the network structure.

The structural connectivity of a neural circuit can generate various functional states. However, not all functional states capable of circuit connectivity are activated. To understand the neural computation of a neuronal circuit, we need to know the relationship between structural connectivity and functional connectivity. The main advantage of *in vitro* experiments is that they are easier to control and relatively better for duplicating results than *in vivo* studies. *In vitro* neuronal circuit is not same as *in vivo* one, but *in vitro* circuits also generate diverse activities, such as various synchronized bursting pattern dynamics or complex oscillatory behavior (Wagenaar et al., 2006; Kim et al., 2014; Kim et al., 2015). Those various network dynamics of neurons were studied, using MEA *in vitro* recording systems, and the studies were limited to population dynamics due to its spatial resolution. To approach better spatial resolution or more organized structures, simple micropatterning has combined with MEA recording systems. The topologies of cultured neurons were controlled by micropatterning and studied differences of them in their activities (Boehler et al., 2012; Marconi et al., 2012). However, understanding the relationship between network structure and its function at a single-neuron resolution has not been reached. To overcome the limitation of recording resolution of MEA systems, the high-density MEA (HDMEA) was developed (Hafizovic et al., 2007). A CMOS based HDMEA allowed subcellular recording resolution (Obien et al., 2014), but still structural analysis of neuronal network at cellular resolution has not been achieved (Ullo et al., 2014). As we already demonstrated combing negative-dot array patterning and a MEA recording system in a previous study (Ryu et al., 2016), our new system, which analyzes a comprehensive neuronal connectome, can be combined with a multi-channel electrophysiological recording system, such as (HD)MEAs, for neuronal populations, providing a new approach to understanding relationships between neuronal structures and circuit function in the future.

In addition, there are general rules for neural circuits across different species, different brain circuits, or even different scales. For example, a hub structure was found at the cellular level as well as in large-scale functional networks of the brain (Bullmore and Sporns, 2009; Cossart, 2014; van den Heuvel and Sporns, 2019). Likewise, we found a scale-invariant characteristic in motif analysis. As described in the results, the class 7 motif (m^3^_7_) of three-node motifs is a rarely preferred structure in the cellular network of neurons (Figure 5) as well as high-order functional networks in the brain (Gollo et al., 2014). Therefore, *in vitro* experiments are typically used to determine the intrinsic properties of (a network of) neurons, used for basic research prior to *in vivo* experiments, or used as an auxiliary tool. The system proposed here is also expected to be useful for monitoring changes in network connectivity through electrophysiological, chemical, or genetic treatments. A neuron can function normally *in vivo* only when the location and growth patterns fit, and if either or both are not suitable, they cannot function properly.

### Limitation of the Study

The accuracy of this new analysis system for neuronal connections is largely dependent on the quality of microcontact printing and multicolor cell labeling. Improvement in patterning with microfluidics (Lee et al., 2018), for example, may improve the quality of patterning. The acuity of tracing individual neurons may also be improved through time-lapse imaging.

This *in vitro* network analysis system cannot determine the ways in which the artificial neuronal network can mimic “real” neural circuits. Though there exists possible differences between the artificial neuronal circuits and brain circuits in not only the neuronal morphology but also their electrophysiological properties (Zhu et al., 2016), the benefits of *in vitro* experimental systems are irrefutable.

## Data Availability Statement

The original contributions presented in the study are included in the article/Supplementary Material, further inquiries can be directed to the corresponding author/s.

## Ethics Statement

The animal study was reviewed and approved by Korea University guidelines and were approved by the Korea University Institutional Animal Care and Use Committee.

## Author Contributions

JK, JR, and WS designed the experiments. JK, JR, and BL performed the experiments. JK performed the analysis. UC, JS, BP, and I-JC fabricated the patterned silicone wafers. JK and WS wrote the manuscript. All authors contributed to the article and approved the submitted version.

## Conflict of Interest

The authors declare that the research was conducted in the absence of any commercial or financial relationships that could be construed as a potential conflict of interest.

## Publisher’s Note

All claims expressed in this article are solely those of the authors and do not necessarily represent those of their affiliated organizations, or those of the publisher, the editors and the reviewers. Any product that may be evaluated in this article, or claim that may be made by its manufacturer, is not guaranteed or endorsed by the publisher.

## Supplementary Material

Supplementary Figure 1 Flowchart of iCANN.

Supplementary Figure 2 Illustrations of Sema3F micropatterning with PDMS stamp on a culture substrate.

Supplementary Figure 3 Time-lapse images of axon and growth cone.

Supplementary Figure 4 Maximal extension of axons from cell bodies. All types of shapes share same area and gap (*D* = 30 μm) between neighboring patterns. Triangle: *L* = 135 μm (*N* = 52). Square: *L* = 89 μm (*N* = 41). Hexagon: *L* = 55 μm (*N* = 46). Adjusted *P* values: Triangle vs. Square 0.0040, Triangle vs. Hexagon < 0.0001, Square vs. Hexagon 0.1396 by one-way ANOVA with post-hoc Tukey.

Supplementary Figure 5 Images of neurons cultured without a pattern **(A)**, and on hexagonal patterns (*D* = 70 μm, *L* = 55 μm) **(B)**. Red: MAP2, Green: Tuj1, Blue: DAPI. Scale bar: 150 μm.

Supplementary Figure 6 Joint probability distribution of six cultured networks.

## Abbreviations

AAV: adeno-associated viruses
DIV: days *in vitro*
FFT: Fast Fourier Transform
HDMEA: high-density multi-electrode array
ICM: incomplete culture media
MEA: multi-electrode array
PBS: phosphate-buffered saline
RPA: receptive pattern area
Sema3F: Semaphorin 3F
TBS: Tris-buffered saline.
