# Supplementary figures and images for: Interpreting the Entire Connectivity of Individual Neurons in Micropatterned Neural Culture With an Integrated Connectome Analyzer of a Neuronal Network (iCANN)
Source: Front Neuroanat. 2021 Oct 20;15:746057. doi: 10.3389/fnana.2021.746057 (PMC8564400; doi:10.3389/fnana.2021.746057)

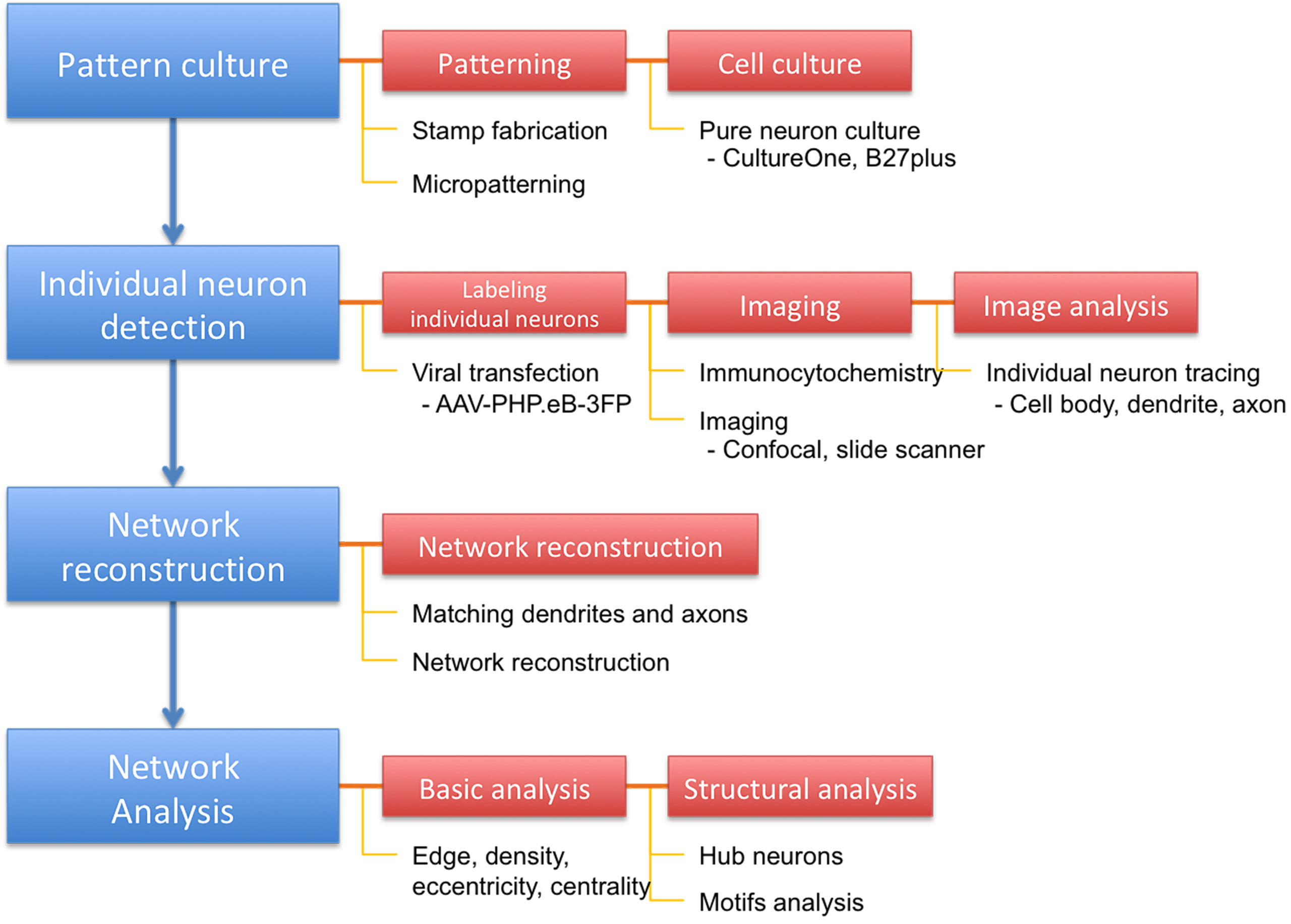

Supplement: Supplementary Figure 1 — Flowchart of iCANN. [file Image_1.PNG]

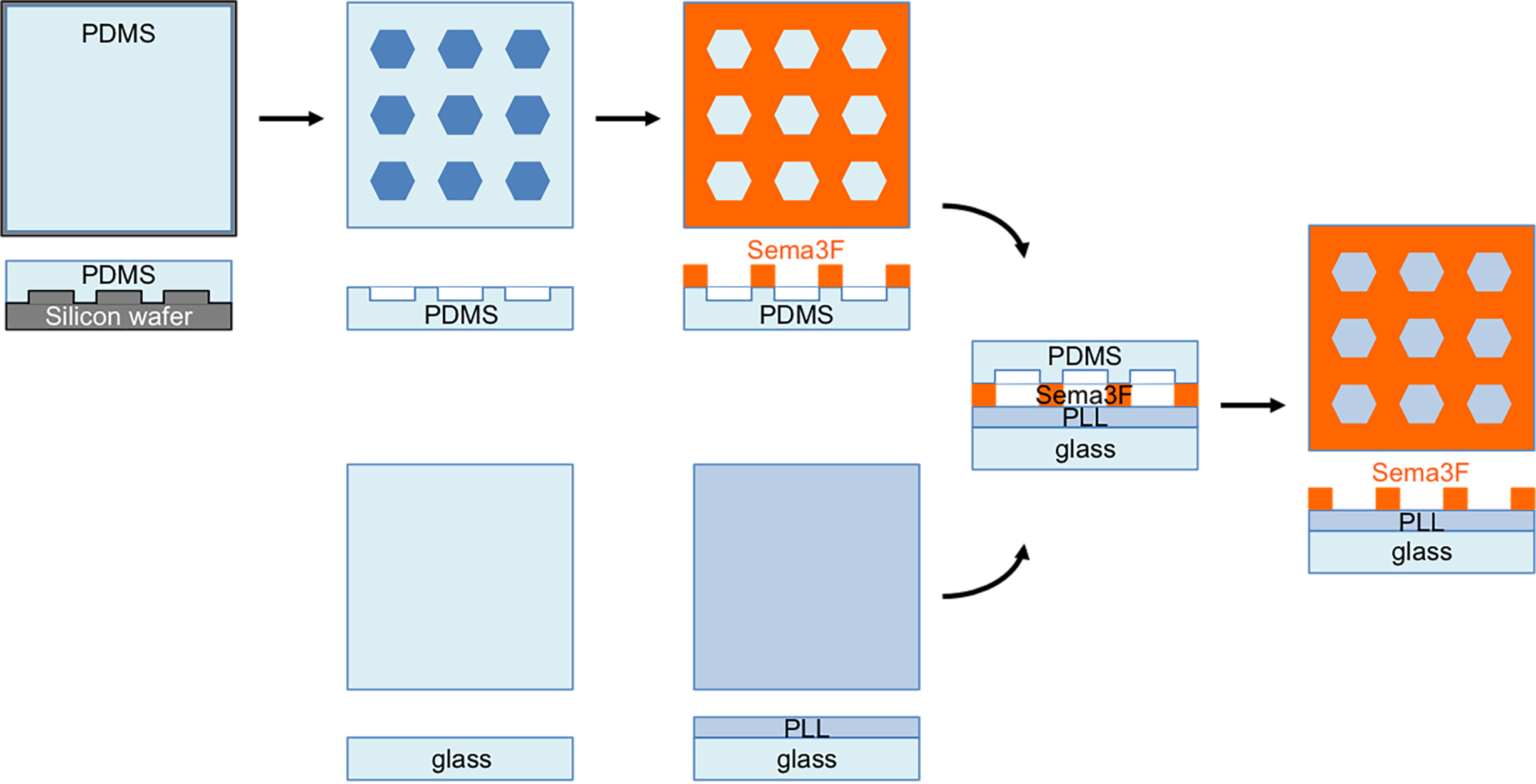

Supplement: Supplementary Figure 2 — Illustrations of Sema3F micropatterning with PDMS stamp on a culture substrate. [file Image_2.PNG]

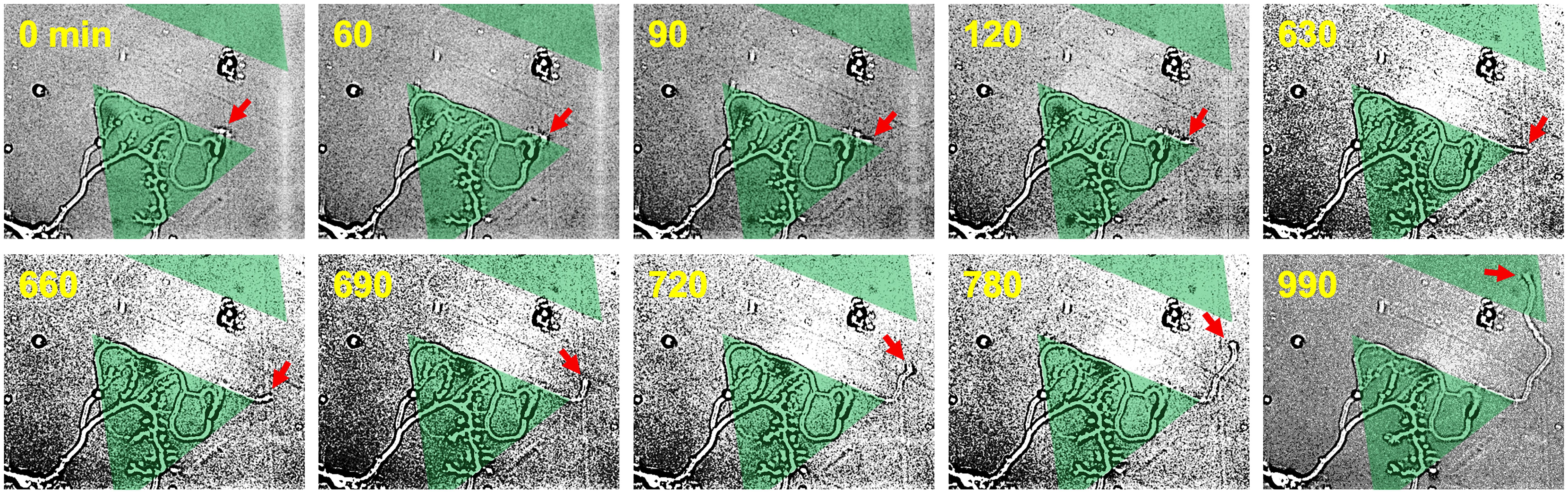

Supplement: Supplementary Figure 3 — Time-lapse images of axon and growth cone. [file Image_3.PNG]

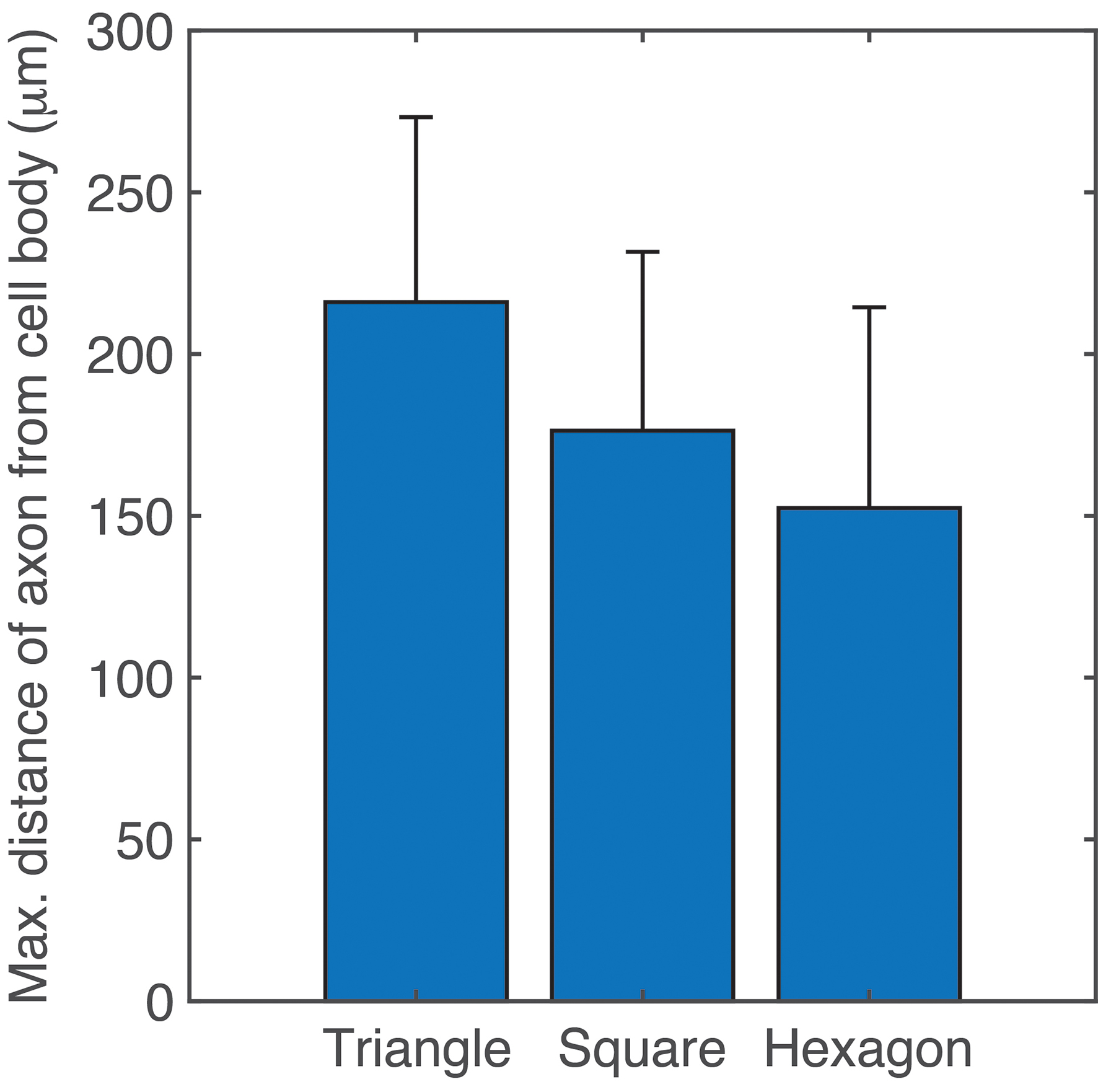

Supplement: Supplementary Figure 4 — Maximal extension of axons from cell bodies. All types of shapes share same area and gap (D = 30 μm) between neighboring patterns. Triangle: L = 135 μm (N = 52). Square: L = 89 μm (N = 41). Hexagon: L = 55 μm (N = 46). Adjusted P values: Triangle vs. Square 0.0040, Triangle vs. Hexagon < 0.0001, Square vs. Hexagon 0.1396 by one-way ANOVA with post-hoc Tukey. [file Image_4.JPEG]

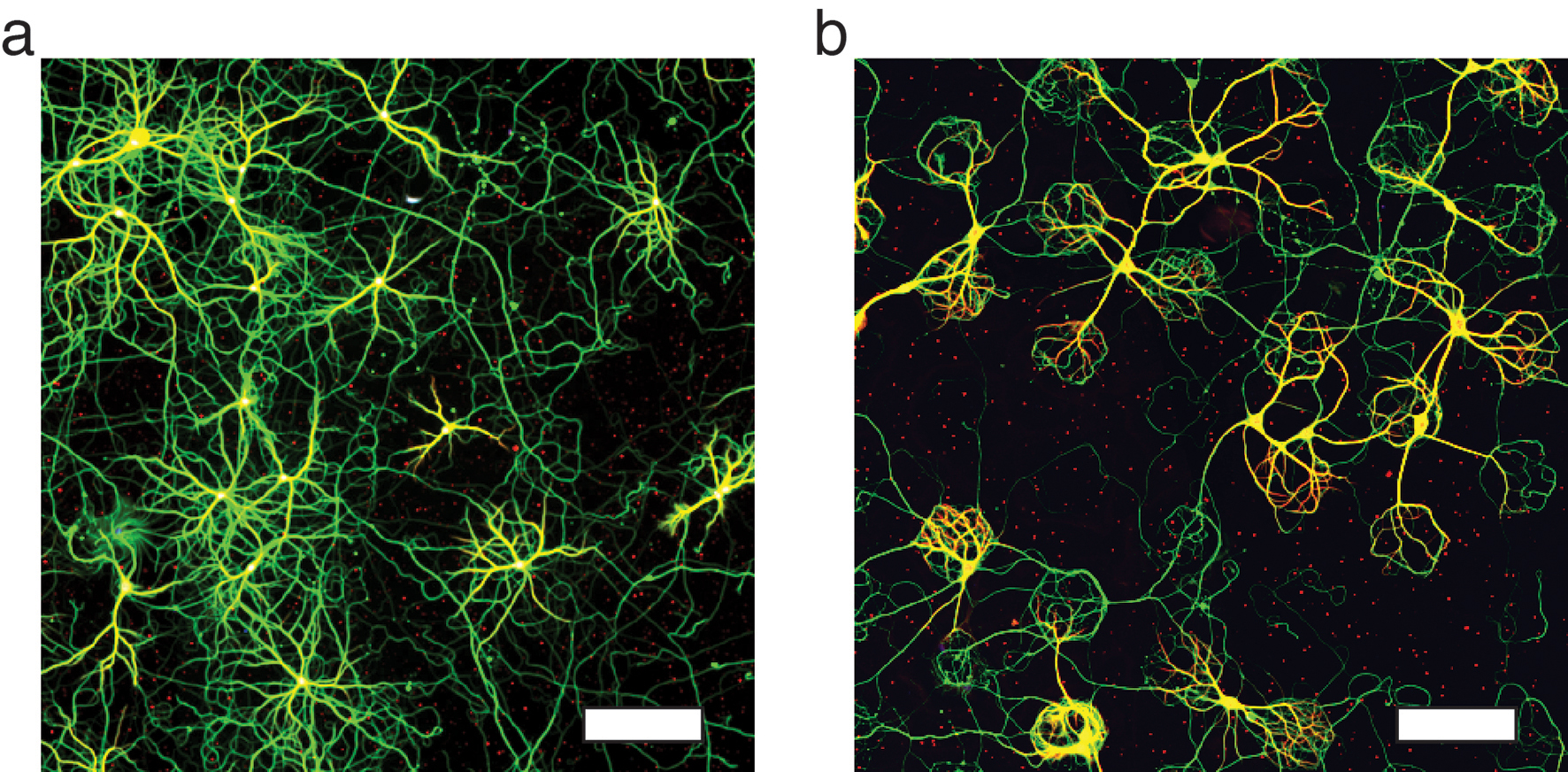

Supplement: Supplementary Figure 5 — Images of neurons cultured without a pattern (A), and on hexagonal patterns (D = 70 μm, L = 55 μm) (B). Red: MAP2, Green: Tuj1, Blue: DAPI. Scale bar: 150 μm. [file Image_5.JPEG]

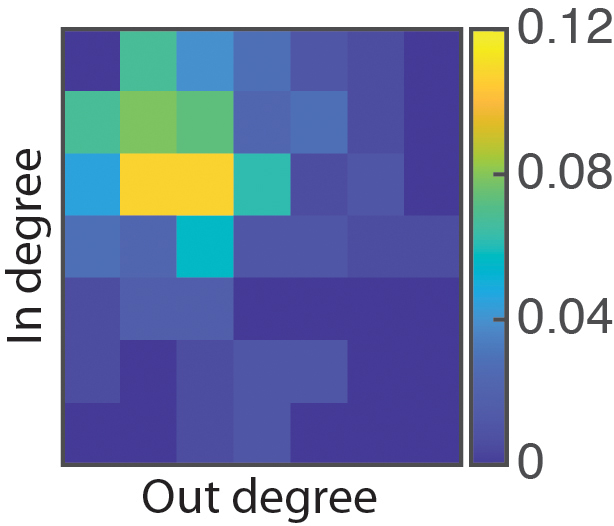

Supplement: Supplementary Figure 6 — Joint probability distribution of six cultured networks. [file Image_6.JPEG]
